# Temporal Dynamics of Norovirus GII.4 Variants in Brazil between 2004 and 2012

**DOI:** 10.1371/journal.pone.0092988

**Published:** 2014-03-25

**Authors:** Julia Monassa Fioretti, Gonzalo Bello, Mônica Simões Rocha, Matias Victoria, José Paulo Gagliardi Leite, Marize Pereira Miagostovich

**Affiliations:** 1 Laboratório de Virologia Comparada e Ambiental, Instituto Oswaldo Cruz – Fiocruz, Rio de Janeiro, Brazil; 2 Laboratório de AIDS & Imunologia Molecular, Instituto Oswaldo Cruz – Fiocruz, Rio de Janeiro, Brazil; 3 Universidad de La República, Laboratorio de Virología Molecular – Regional Norte, Ciudad de Salto, Uruguay; CEA, France

## Abstract

Noroviruses (NoVs) are the major cause of acute gastroenteritis outbreaks, and, despite a wide genetic diversity, genotype II.4 is the most prevalent strain worldwide. Mutations and homologous recombination have been proposed as mechanisms driving the epochal evolution of the GII.4, with the emergence of new variants in 1–3-year intervals causing global epidemics. There are no data reporting the dynamics of GII.4 variants along a specific period in Brazil. Therefore, to improve the understanding of the comportment of these variants in the country, the aim of this study was to evaluate the circulation of NoV GII.4 variants during a 9-year period in 3 out of 5 Brazilian regions. A total of 147 samples were sequenced, and a phylogenetic analysis of subdomain P2 demonstrated the circulation of six GII.4 variants, Asia_2003, Hunter_2004, Den Haag_2006b, Yerseke_2006a, New Orleans_2009, and Sydney_2012, during this period. The most prevalent variant was Den Haag_2006b, circulating in different Brazilian regions from 2006 to 2011. A Bayesian coalescent analysis was used to calculate the mean evolutionary rate of subdomain P2 as 7.3×10^−3^ (5.85×10^−3^–8.82×10^−3^) subst./site/year. These analyses also demonstrated that clade Den Haag_2006b experienced a rapid expansion in 2005 and another in 2008 after a period of decay. The evaluation of the temporal dynamics of NoV GII.4 in Brazil revealed a similar pattern, with few exceptions, to the worldwide observation. These data highlight the importance of surveillance for monitoring the emergence of new strains of NoV GII.4 and its impact on cases of acute gastroenteritis.

## Introduction

Noroviruses (NoVs) are considered to be the major causative agent of acute gastroenteritis (AGE) outbreaks worldwide [1]. In developing countries, is estimated that it causes annually 1.1 million hospitalizations and 200,000 deaths in children under 5 years of age [2].

The genus *Norovirus* belongs to the Caliciviridae family, which comprises nonenveloped viruses with an icosahedral symmetry of approximately 27–30 nm in diameter. The NoV genome is composed of a positive-sense single-stranded RNA covalently linked to VPg at the 5′ end and polyadenylated at the 3′ end, 7.5–7.7 kb in length. The genome is organized into three open reading frames (ORF), with ORF1 encoding six non-structural proteins, including the RNA-dependent RNA-polymerase, ORF2 encoding the structural protein VP1 that composes the viral capsid, and ORF3 encoding the structural protein VP2 [3]. VP1 is divided into a highly conserved shell (S) domain and a variable protruding (P) domain, which is further subdivided into subdomains P1 and P2. The protruding P2 domain, located at the most exposed surface of the viral capsid, is the major responsible for the antigenicity of the virus and determines interaction with host cell attachment factors (histo-blood group antigen, HBGA) [4], [5]. NoV has been classified into five genogroups on the basis of the VP1 sequence (GI to GV) that can be further subdivided into more than 36 genotypes [6]. Recently, a standardization of NoV nomenclature was proposed based on the classification of the VP1 and ORF1 sequences due to the common event of recombination in the hotspot ORF1/ORF2 [7].

Despite the wide genetic diversity of NoV, GII.4 has been described as the most prevalent NoV genotype and has been associated with AGE outbreaks and sporadic cases worldwide since the mid-1990s [8], [9], [10], [11]. Two mechanisms have been proposed to allow the persistence of NoV GII.4 in the population: receptor switching and antigenic variation, which facilitate an expansion of the host range and the escape of new strains from herd immunity [12]. Many studies have shown that the rapid rate of evolution and fixation of amino acid changes in the capsid P2 subdomain might result in altered antigenicity, allowing escape from the host immune responses directed toward the previous circulating variant [10], [13], [14], [15]. This continuous diversification allows the emergence of new epidemic NoV GII.4 lineages in incremental periods, a temporal dynamic comparable to that of influenza A virus [16].

The first GII.4 variant associated with epidemic outbreaks was 95/96US detected in Australia, Europe, and the USA [17], [18]. Since this time, six pandemic variants named Farmington Hills_2002, Hunter_2004, Yerseke_2006a, Den Haag_2006b, New Orleans_2009, and Sydney_2012 have been described as emerging in intervals of 1–3 years [9], [14], [15], [19]–[27]. Other GII.4 variants have been described, including Henry_2001, Japan_2001, Asia_2003, Osaka_2007, and Apeldoorn_2008; however, these viruses exhibited no pandemic characteristics [22], [28]–[31]. Hence, the constant surveillance of new emerging variants of NoV GII.4 is fundamental due to its large impact on public health [32].

Brazilian studies regarding NoV epidemiology refer to the characterization of samples from a single state or geographic region, whereas there is an absence of large-scale analyses that meet the continental dimensions of the country [11], [33]–[41]. Recently, our group demonstrated the genetic diversity of NoV genotypes circulating in different Brazilian regions [42]. However, the specific GII.4 variants circulating in Brazil remain unknown. Thus, the present study aims to expand the data regarding the temporal dynamics and genetic characteristics of NoV GII.4 variants circulating in the country between 2004 and 2012.

## Materials and Methods

### Sample Collection

Stool samples were obtained by spontaneous demand of inpatients and outpatients from AGE outbreaks or sporadic cases attending in public health centers including hospitals and central laboratories of the country. Samples were shipped to the laboratory under refrigeration for the diagnosis of viral gastroenteritis. Between 2004 and 2012 the laboratory tested 8900 stool samples for NoV diagnosis, confirming 30.1% of those. The percentage of annual detection ranged from 19.5% to 32.7%, except in 2006 when this percentage reached 47.4% (659/1389). NoV were detected using the region B (RNA-dependent RNA-polymerase) RT-PCR protocol previously described [43] and genotyped as NoV GII.4 by the partial nucleotide sequencing of region D (VP1) [44]. A total of 147 NoV GII.4 samples previously characterized were selected from 12 states of the three most highly populated regions of Brazil (Northeast, South, and Southeast), representing approximately 85% of the countrýs population (190.732.694 inhabitants - Brazilian Institute of Geography and Statistics - IBGE 2010).

### Ethics Statement

This study was approved by the Ethics Committee of Oswaldo Cruz Foundation (CEP 311/06) and is part of an official Brazilian Ministry of Health’s surveillance. In this ongoing program the diagnosis of gastroenteritis is required to elucidate the viral etiology and data are maintained anonymous.

### Molecular Characterization

A 10% stool suspension was prepared in 0.01 M Tris-HCl and 0.0015 M Ca^2+^ (pH 7.2). The RNA extraction was performed using a QIAmp viral RNA Mini kit (Qiagen, Valencia, Calif., USA) according to the manufacturer’s instructions, and the synthesis of complementary DNA (cDNA) was performed using a random primer, pd(N)6 (Amersham Biosciences, UK). The amplification was performed using a set of primers that target the partial region of the genome that encodes the main VP1 capsid protein (subdomain P2), a fragment situated in ORF2 [23]. The amplicons obtained were purified using the QIAquick PCR Purification Kit (QIAGEN, Valencia, CA, USA) following the manufacturer’s recommendations and quantified using a spectrophotometer (Qubit, USA). DNA sequencing was performed using the dideoxynucleotide chain termination method with the ABI Prism Big Dye Terminator Cycle Sequencing Ready Reaction Kit 1, v. 3.1 and the ABI Prism 3730 Genetic Analyzer (both from Applied Biosystems, Foster City, CA, USA) by the genomic platform of DNA sequencing PDTIS/FIOCRUZ.

### Phylogenetic Analyses

The Brazilian sequences obtained in this study were aligned with reference strains of each NoV GII.4 variant and three sequences from Taiwan (HQ456344), Sweden (JN183166), and Japan (AB541348) using the tool CLUSTALW in the MEGA5.2 program [45], [46]. A Maximum Likelihood (ML) tree was reconstructed with program PhyML [47] using an online web server [48]. A heuristic tree search was performed using the SPR branch-swapping algorithm, and the reliability of the obtained topology was estimated with the approximate likelihood-ratio test (*aLRT*) [49] based on a Shimodaira-Hasegawa-like procedure. The trees were visualized using the FigTree v1.3.1 program [50].

### Evolutionary and Demographic Analyses

The rate of nucleotide substitution per site per year (subs./site/year), the time to the most recent common ancestor (T_MRCA_), and the demographic history of NoV GII.4 were jointly estimated using the Bayesian Markov chain Monte Carlo (MCMC) approach implemented in the BEAST v1.7.5 package [51]. The temporal scale of the evolutionary process was directly estimated from the sampling dates of the sequences using the GTR+I+Г_4_ nucleotide substitution model, selected using the jModeltest program [52], a relaxed uncorrelated lognormal molecular clock model [53], and a Bayesian Skyline coalescent tree prior [54]. The MCMC analysis was performed for 50 million generations, and the convergence of parameters was assessed by calculating the Effective Sample Size (ESS) using the TRACER v1.5 program after excluding the initial 10% of the run. Uncertainty in the parameter estimates was reflected in 95% HPD intervals. The programs TreeAnnotator v1.7.5 and FigTree v1.4.0 were used to summarize the posterior tree distribution and to visualize the annotated Maximum Clade Credibility (MCC) tree, respectively.

### GenBank Accession Numbers

The nucleotide sequences obtained in this study were submitted to the National Center for Biotechnology Information (GenBank, http://www.ncbi.nlm.nih.gov/) and received the following accession numbers: JX975499–JX975614 and KF434555–KF434585.

## Results

### Identification of NoV GII.4 Variants Circulating in Brazil

**Figure 1:**
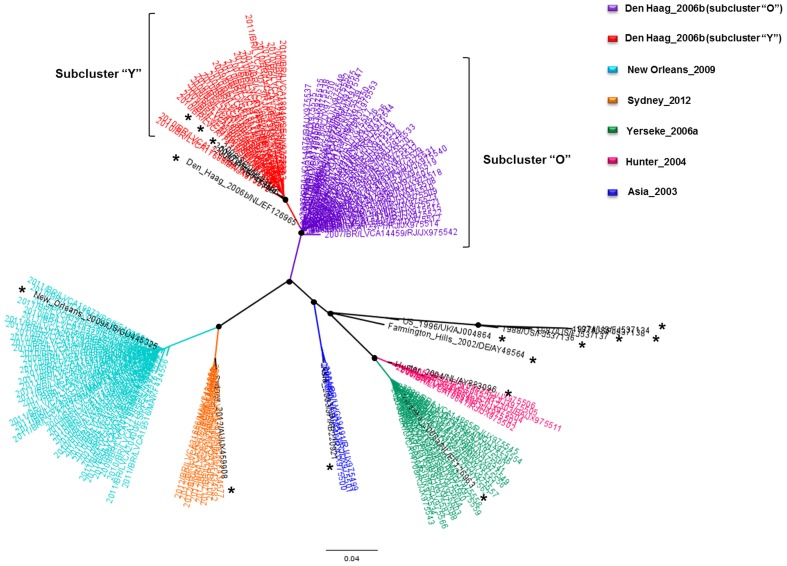
ML tree of P2 subdomain sequences (630 pb) from Brazilian (n = 147) and non-Brazilian strains (n = 15). The black circles represent nodes with aLRT ≥0.9. The non-Brazilian samples are marked in black and with an asterisk. Horizontal branch lengths are drawn to scale with the bar at the bottom indicating nucleotide substitutions per site.

An ML phylogenetic analysis of the subdomain P2 of gene encoding VP1 from 147 NoV GII.4 Brazilian samples demonstrated the presence of six variants in different states of the country during the 2004–2012 period (Fig. 1). The samples classified as Den Haag_2006b were grouped into two remarkably separate subclusters, here denominated as “O” (oldest), which comprises 40 samples collected during 2006–2008, and “Y” (youngest), which comprises 24 samples from the period 2009–2011 (Fig. 1).

**Figure 2:**
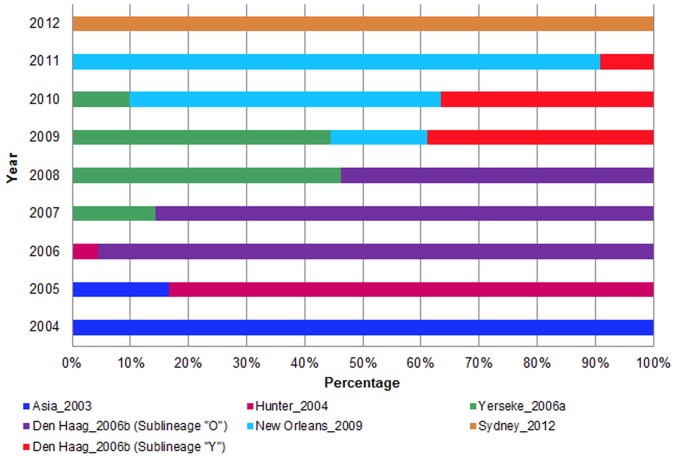
Temporal dynamics of the prevalence of NoV GII.4 variants in Brazil during the 2004–2012 period.

For most of the years analyzed, it was possible to detect at least two different variants co-circulating in Brazil (Table 1 and Fig. 2), and a clear variation in the frequency of the different variants over time was also observed. The most prevalent variants were Asia_2003 in 2004, Hunter_2004 in 2005, Den Haag_2006b in 2006–2010, Yerseke_2006a in 2008–2009, New Orleans_2009 in 2010–2011, and Sydney_2012 in 2012 (Table 1 and Fig. 2). In addition, the variants Den Haag_2006b, Yerseke_2006a, New Orleans_2009, and Sydney_2012 were detected in all three Brazilian regions evaluated.

**Table 1:** NoV GII.4 variants detected in Brazil during the period 2004–2012.

Year	Brazilian region	GII.4 variant
2004	Southeast	Asia_2003 (*n* = 2)
2005	Southeast	Asia_2003 (*n* = 1); Hunter_2004 (*n* = 5)
2006	Northeast, South, Southeast	Hunter_2004 (*n* = 1); Den Haag_2006b (*n* = 21)
2007	Northeast, South, Southeast	Yerseke_2006a (*n* = 2); Den Haag_2006b (*n* = 12)
2008	Northeast, South, Southeast	Yerseke_2006a (*n* = 6); Den Haag_2006b (*n* = 7)
2009	Northeast, South, Southeast	Yerseke_2006a (*n* = 8); Den Haag_2006b (*n* = 7);
		New Orleans_2009 (*n* = 3)
2010	Northeast, South, Southeast	Yerseke_2006a (*n* = 4); Den Haag_2006b (*n* = 15);
		New Orleans_2009 (*n* = 22)
2011	Northeast, South, Southeast	Den Haag_2006b (*n* = 2); New Orleans_2009 (*n* = 20)
2012	Northeast, South, Southeast	Sydney_2012 (*n* = 9)

The number in parentheses denotes the quantity of samples sequenced.

### Evolutionary History of NoV GII.4 Variants

**Figure 3:**
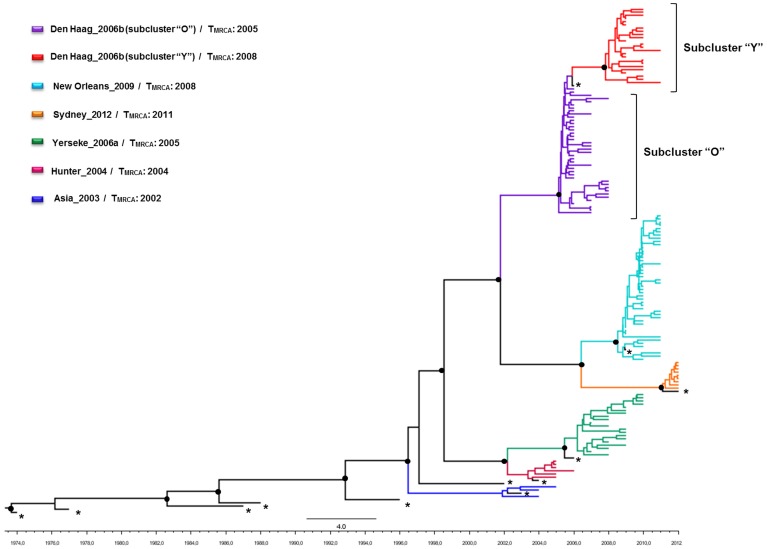
MCC tree of NoV GII.4 subdomain P2 sequences (630 pb) from Brazilian (n = 147) and non-Brazilian strains (n = 12). The black circles represent nodes with posterior ≥0.9. The non-Brazilian samples are marked in black and with an asterisk.

To reconstruct the time-scale of the NoV GII.4 epidemics, we performed a Bayesian coalescent analysis of partial VP1 sequences of Brazilian origin and reference samples isolated between 1974 and 2012. The mean evolutionary rate of the subdomain P2 was calculated as 7.3×10^−3^ (5.85×10^−3^–8.82×10^−3^) subst./site/year. Considering this substitution rate, the mean T_MRCA_ of the NoV GII.4 variants were estimated as follows: 2002 (95% HPD: 2001–2003) for Asia_2003; 2003 (95% HPD: 2003–2004) for Hunter_2004; 2005 (95% HPD: 2005–2006) for Yerseke_2006a; 2005 (95% HPD: 2004–2006) for Den Haag_2006b; 2008 (95% HPD: 2008–2009) for New Orleans_2009; and 2011 (95% HPD: 2011–2012) for Sydney_2012 (Fig. 3). Within the Den Haag_2006b variant, the T_MRCA_ of subclusters “O” and “Y” was dated to 2005 (95% HPD: 2005–2006) and 2008 (95% HPD: 2007–2008), respectively.

### Demographic History of the NoV GII.4 Den Haag_2006b Variant

**Figure 4:**
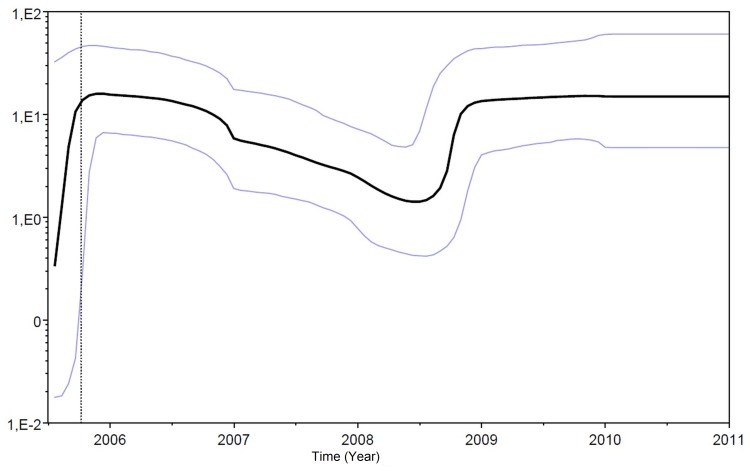
Bayesian skyline plot of variant Den Haag_2006b. The black line represents the median posterior value, and the blue lines indicate the 95% Highest Probability Density (HPD) intervals. The Y-axis depicts the value of Net on a logarithmic scale.

Estimations of the demographic history of the NoV GII.4 Den Haag_2006b lineage was next performed using the Bayesian skyline plot method (Fig. 4). This analysis demonstrated that clade Den Haag_2006b experienced a rapid expansion in 2005 after which the population reached a maximum effective population size in early 2006 and then began to decay. It is possible to observe a new and sharp expansion of the effective population size circa mid-2008, which coincides with the T_MRCA_ of subcluster “Y”, and a later stabilization during 2009 and 2010.

## Discussion

NoV GII.4 is the most prevalent genotype of NoV is associated with worldwide acute gastroenteritis outbreaks [10], [11], [14], [42], [55]. A phylogenetic analysis of subdomain P2 of the VP1 gene allowed the identification of six different variants of NoV GII.4 that were sequentially detected in Brazil during the 2004–2012 periods. In most of the years analyzed, two or three different GII.4 variants were detected simultaneously in Brazil, a pattern comparable to that observed in Japan during the years 2005–2009 [22].

The first variant detected in Brazil in 2004 was Asia_2003, which was described as a recombinant strain, with an ORF1 sequence belonging to GII.12 (Wortley-like) and ORF2 and −3 sequences belonging to GII.4. However, this variant does not feature pandemic characteristics and was demonstrated as only causing outbreaks in Asia, Oceania, and the United Kingdom between 2002 and 2006 [14], [30], [56], [57]. The lineage Hunter_2004 was the next to become dominant in Brazil in 2005 and was associated with global gastroenteritis outbreaks between 2004 and 2005 [58]. The first description of this variant in Latin America was in Paraguayan children also during the years 2004 and 2005 [58].

Two different variants most likely emerged in 2005 and were detected globally in 2006: the Yerseke_2006a and Den Haag_2006b variants that share a common ancestor with Hunter_2004 and Fargminton Hills_2002, respectively. Both variants were detected in all Brazilian regions analyzed, but they spread and died out with very different dynamics. The Den Haag_2006b variant replaced Hunter_2004 in Brazil in 2006 and was the most prevalent lineage during the period of 2006–2008. The Yerseke_2006a variant was first detected in Brazil in 2007 and did not replace the previous variant Den Haag_2006b, though both variants co-circulated at equal proportions during 2008–2009. The detection rate of Yerseke_2006a decreased by 2010, whereas Den Haag_2006b remained detectable until 2011. Another different temporal dynamic was observed in Europe and Oceania, where Yerseke_2006a was the dominant clade in the 2005/2006 season and was replaced by Den Haag_2006b in summer/fall of 2007 [60], [61], [62].

The variant New Orleans_2009 was first detected to be circulating in Brazil in December 2009. By 2010, this variant reached a frequency that was similar to variants Yerseke_2006a and Den Haag_2006b together, becoming the dominant clade by 2011. In the USA, this variant was first detected during the winter of 2009/2010, causing 60% of all outbreaks associated with NoV and replacing the Den Haag_2006b variant as the predominant GII.4 lineage [23], [24].

The variant Sydney_2012 was first detected in 2012 in all of the Brazilian regions evaluated - Northeast, Southeast, and South - replacing New Orleans_2009. A recent report demonstrated this variant as circulating in Northern Brazil, causing sporadic cases in hospitalized children [41]. This strain also became the predominant NoV variant in the USA in 2012/2013, but its emergence was not associated with an increase in the activity of outbreaks when compared to previous seasons [63].

The mean estimated rate of evolution (7.3×10^−3^ subst./site/year) of subdomain P2 of VP1 in our study was similar to the rates reported in previous studies for the entire VP1 gene (1.9 to 9.0×10^−3^ subst./site/year) [15], [64], [65], [66]. The mean T_MRCA_ of the six variants detected in Brazil [Asia_2003 (2002), Hunter_2004 (2003), Yerseke_2006a (2005), Den Haag_2006b (2005), New Orleans_2009 (2008), and Sydney_2012 (2011)] demonstrates that the diversification process starts at least one year before the variant becomes a dominant strain and causing epidemics worldwide. Many studies have proposed that a strain circulates at low levels, accumulating mutations for some time before became pandemic [62].

The reconstruction of the demographic history of the Den Haag_2006b clade demonstrates that, after a rapid increase in 2005, the population reached a maximum effective size early in 2006 (when this variant was first detected) and then decayed until mid-2008. Since mid-2008, the Den Haag_2006b lineage sharply increased again, reaching a new maximum effective population size in 2009. A recent Argentinian study evaluated the temporal dynamics of this variant and found that the effective population size increased in mid-2004 and started to decrease in 2008 [67]. Such complex demographic patterns of this variant might be related to the existence of two distinct sublineages of Den Haag_2006b: the “Y” sublineage that emerged circa 2008 is a descendant from the “O” sublineage; from most likely emerged circa 2005. The estimated emergence of the subcluster “Y” coincides with the second expansion phase of the Den Haag_2006b variant. The identification of a few samples from Taiwan, Sweden, and Japan that grouped with subcluster “Y” suggests that this sublineage is not exclusive to Brazil. It is interesting to note that subcluster “Y” displayed a large number of substitutions at residues 255, 357, 368 and 425 in relation to subcluster “O” (data not shown). The residue 368 is located in putative epitope “A” of the viral capsid that is most likely responsible for the difference in antigenicity among NoV GII.4 variants [68], [69], thus suggesting that subclusters “O” and “Y” of the Den Haag_2006b lineage may represent distinct antigenic variants of NoV GII.4.

As suggested by de Rougemont et al. (2011) [70], the greater prevalence of variant Den Haag_2006b could correlate with the binding capability to secretor and nonsecretor populations when compared with Yerseke_2006a strains. In Brazil, there is only one study that characterizes the genetic diversity of the HBGAs types from a small population of descendants of slaves living in semi-isolated rural areas [71]. Further studies including structure modeling of these variants and characterization of HBGAs polymorphisms of Brazilian population to relate the binding properties of NoV should be conducted for clear understanding of predominance of variants GII.4 in the country.

In developed countries, the emergence and replacement of NoV GII.4 variants over the years have pointed to the establishment of NoV surveillance networks as an approach to a better understanding of the epidemiology of this virus and to guide preventive measures against outbreaks [23], [32]. In the present study, the temporal dynamics of NoV GII.4, with few exceptions, were similar worldwide, stressing the need for further studies on the continuous epidemiological surveillance of the circulation of NoV genotypes and GII.4 variants in Brazil.
